# The gut wall provides an effective barrier against nanoparticle uptake

**DOI:** 10.3762/bjnano.5.218

**Published:** 2014-11-12

**Authors:** Heike Sinnecker, Thorsten Krause, Sabine Koelling, Ingmar Lautenschläger, Andreas Frey

**Affiliations:** 1 Division of Mucosal Immunology & Diagnostics, Priority Program Asthma & Allergy, Research Center Borstel, Leibniz Center for Medicine and Biosciences, Parkallee 22, Borstel, 23845, Germany, Airway Research Center North (ARCN), Member of the German Center for Lung Research; 2 Central Laboratory of Analytical Chemistry, Hamburg University of Technology, Eißendorfer Straße 38, Hamburg, 21073, Germany; 3 Department of Anesthesiology and Intensive Care Medicine, University Hospital Schleswig-Holstein, Schwanenweg 21, Kiel, 24105, Germany

**Keywords:** barrier, isolated intestine, mucus, nanoparticles, uptake

## Abstract

**Background:** The omnipresence of nanoparticles (NPs) in numerous goods has led to a constant risk of exposure and inadvertent uptake for humans. This situation calls for thorough investigation of the consequences of NP intake. As the vast mucosa of the human gastrointestinal tract represents an attractive site of entry, we wanted to take a look on the fate that ingested NPs suffer in the gut. As a model to investigate NP uptake we used the isolated perfused rat small intestine. Differently sized fluorescent latex particles were used as exemplary anthropogenic NPs.

**Results:** The particles were administered as bolus into the isolated intestine, and samples from the luminal, vascular and lymphatic compartments were collected over time. NP amounts in the different fluids were determined by fluorescence measurements. No particles could be detected in the vascular and lymphatic system. By contrast a major amount of NPs was found in luminal samples. Yet, a substantial share of particles could not be recovered in the fluid fractions, indicating a sink function of the intestinal tissue for NPs. A histological examination of the gut revealed that virtually no particles adhered to the epithelium or resided in the tissue, the bulk of particles seemed to be trapped in the mucus lining the gut tube. When this mucus was dissolved and removed from the gut almost the entire amount of particles missing could be recovered: over 95% of the given NPs were present in the two fractions, the luminal samples and the dissolved mucus. To foster NP uptake via an extended interaction time with the epithelium, the intestinal peristalsis was decelerated and the duration of the experiment was prolonged. Even under those conditions, no particle fluorescence was detected in the vascular and lymphatic samples.

**Conclusion:** We could show that after intestinal exposure with a large dose of NPs the vast majority of NPs did obviously not come into contact with the epithelium but was either directly discarded from the gut or trapped in mucus. The healthy small intestinal tract evidently provides an effective barrier against NP uptake whereby the mucus film seems to play an important role.

## Introduction

To the same extent that nanoparticles (NPs) find their way into foods, drugs, cosmetic articles and other consumer products [1–3], incorporation of anthropogenic NPs, be it through inhalation or ingestion, becomes an issue. The public is increasingly worried about potential hazards such consumer good-borne NPs may pose to human health [4–6]. Besides that, work environments with metal grinders or welding machines entail exposure to high particle concentrations in the air [7–8]. Adverse effects on lung function to workers in such environments have already been reported [9–11]. In addition, one should keep in mind that inhaled particles are often coughed up and then swallowed and, hence, can be absorbed via the gastrointestinal tract. This is in line, e.g., with a case report in which after chronic inhalation of carbon NPs from toner dust, no respiratory symptoms were reported, but deposition of carbon NPs in the peritoneum were found and the person developed weight loss and diarrhea [12]. In light of this, thorough investigations of the interaction of NPs with the digestive tract are required. With the vast area of 200 m^2^ mucosal surface arranged in circular folds, villi, crypts and microvilli, the small intestine as main place of nutrient resorption [13] represents an attractive site for NP entry.

In the late 1990s, diverse animal feeding studies were conducted in order to quantify the amount of particles ranging from 50 nm to 20 µm in size that is taken up at different mucosal sites, such as the lymphoid- (Peyer’s patches) and non-lymphoid-associated tissue, of the digestive tract. It was found that particles can indeed be translocated, but the efficiency of particle uptake is both, a tissue- and particle size-dependent event. Smaller particles, i.e., less than or equal to 100 nm in diameter, are absorbed more frequently than particles of 500 nm up to 6 µm in size, and more particles were found in gut-associated lymphoid than in non-lymphoid tissues [14–17]. Yet, the investigation of in vivo NP uptake after oral exposure is complicated by many factors which may influence the outcome: dietary status, mucosal secretions and their composition, variabilities in gastric and intestinal pH, gastrointestinal transit time and the gastrointestinal flora [18].

The actual physical mucosal barrier is based on the columnar epithelium of enterocytes lining the luminal surface of the gut and may be described as a combination of individual fencings [19]. The epithelial cells are covered by the glycocalyx, a dense mesh of glycostructures [20], and mucus, a lubricant and gel-like diffusion barrier, is constantly released by goblet cells [21]. If these first lines of defense are penetrated the trans- and paracellular way is strictly controlled by the enterocytes which are connected to each other by tight junctions [22]. The epithelial turnover of the enterocytes provides an additional type of hurdle [23] as NP-ladden intestinal epithelial cells would be sloughed off and be excreted within a few days. For the uptake of supramolecular entities, in particular microbial foes, specialized cell types such as M cells or dendritic cells exist at certain mucosal sites [24–25]. These gateways, intended for the delivery of antigenic matter to the mucosal immune system, may be hijacked by anthropogenic NPs leading to an accumulation of NPs in gut-associated lympoid tissue as indicated by the early studies mentioned above.

Because the native fencings and gateways should be preserved best in native tissue, we decided to use the isolated perfused rat small intestine, recently established in our institute [26], as a model to investigate NP interaction with the digestive tract. In this model the structural barrier of a healthy gut is retained without the complexity of a whole animal model or the simplifications of a mere cell culture system. Our results obtained with this ex vivo system show that the multiple fencings of the intestinal mucosa combine into an effective barrier against NP uptake.

## Results

Nanoparticles, if degradation resistant, ought to behave as individual entities, each one resembling a single, oversized macromolecule. In this respect even high particle numbers translate into very low molarities and molar amounts, e.g., 10^12^ NPs would represent only about a picomole of particulate matter. For tracing of such minute amounts reduction of dilutory effects before and after uptake may be a prerequisite.

**Figure 1:**
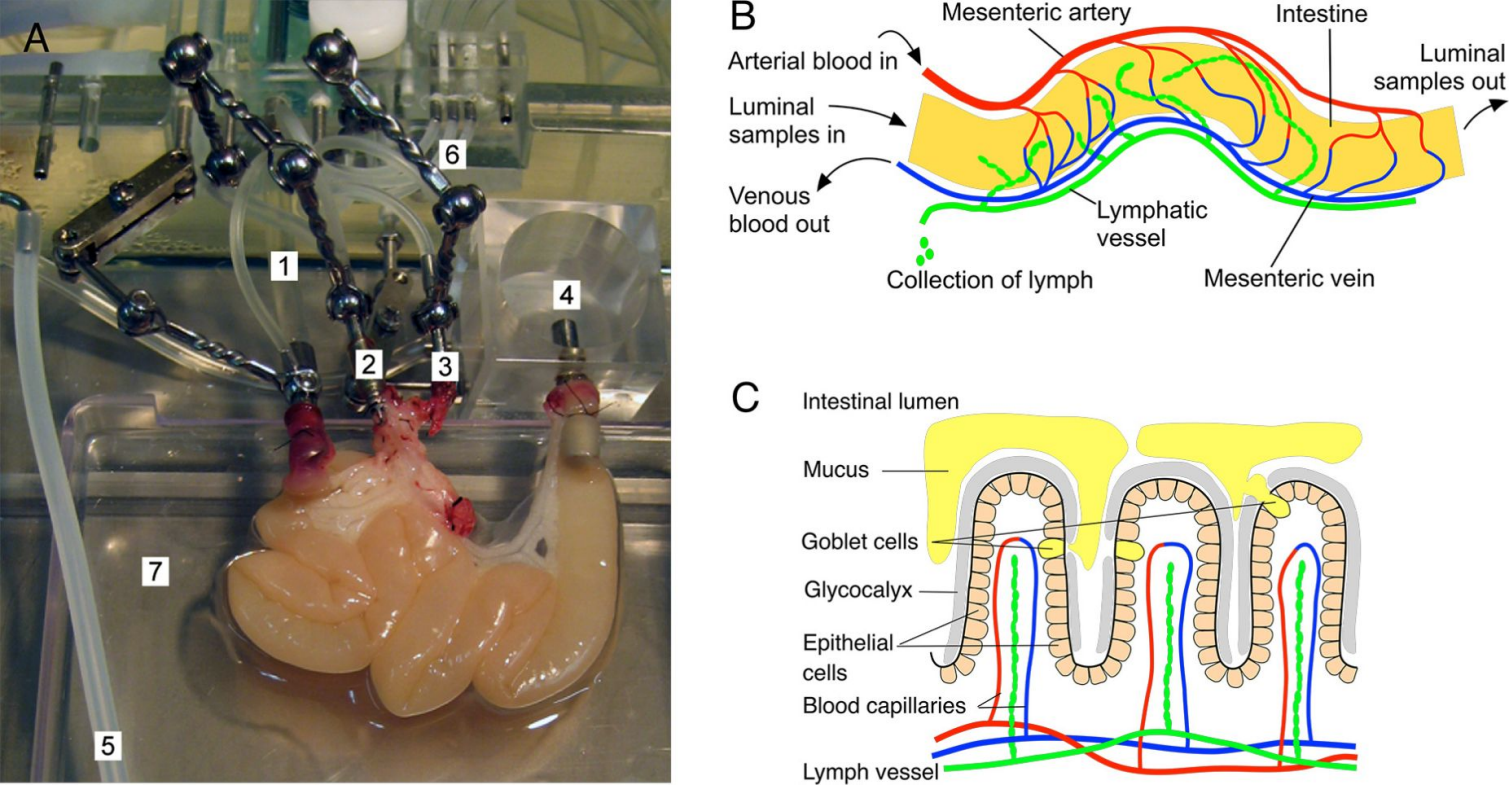
Overview of the intestinal features relevant to the experimental setup. A: The rat intestinal explant in the perfusion chamber. 1: Inlet of luminal buffer and samples, 2: outflow of the portal vein, 3: inlet of artificial blood plasma (vascular buffer) into the mesenteric artery, 4: outflow of luminal content (luminal samples), 5: outflow of lymph (lymphatic samples), 6: tubing to pressure recorders (luminal, blood vessels), 7: electronic balance to check gut weight during the experiment. B: Schematic model of the rat small intestinal explant. Via the cannulated artery the explant is supplied with artificial blood plasma. The lymph vessel is scarified and the outflow aspirated. Particle samples applied luminally are transported through the gut with the slow-going flow of the luminal buffer, and the outflow of the luminal content is collected in fractions. C: Schematic overview of relevant components of the intestinal wall. The tight layer of epithelial cells lining the villous architecture of the gut wall are covered by a network of glycostructures (glycocalyx) and by the mucus which is secreted by goblet cells.

In order to meet this goal and keep a lifelike setting we chose the isolated perfused rat intestine model [26] for our NP uptake studies and modified it according to our specific needs (Figure 1): For improving the detection of even minute amounts of NPs in the vascular compartment, the supply with oxygen and nutrients via artificial blood plasma was changed from a single-path to a recirculating system. Therewith, the amount of artificial plasma could be reduced by factors of 4.5 and 6 for, respectively, 270 and 360 min duration of the experiment. For evaluating the influences of gut motility on particle uptake, the peristalsis was varied by application of different doses of noradrenaline.

Viability of the gut during and after the extracorporal perfusion was monitored over the entire experiment by recording several key parameters. Oxygen and carbon dioxide partial pressure and pH in the arterial and venous compartment stayed within their normal physiological ranges at all times (data not shown). As a measure of aerobic metabolism and metabolic competence the lactate-to-pyruvate ratio and galactose resorption (from digestion of luminal lactose) were determined, both parameters were in comparable physiological ranges, regardless of whether or not NPs were administered (control experiments). Tissue integrity was checked after the experiment by histological examination and always found to be in an acceptable range, with villi and enterocytes remaining intact after 270 or 360 min of perfusion, both in experiments with and without NP application as well as after contact with a reducing agent (see below).

In order to be able to track even rare translocation events, and in light of the huge dilution which the particles were prone to undergo if reaching the vascular system, we decided to use high doses of NPs and to instill them into the gut lumen as a highly concentrated bolus. As anthropogenic model NPs we chose fluorescent, carboxylate-functionalized polystyrene particles of three different sizes (20, 40 and 200 nm) (Table 1).

**Table 1:** Nanoparticles used in the uptake studies with the isolated perfused rat intestine.

name of NP	label	size [nm]^a^	administered NP (bolus)	detection limit [NP/mL]^b^

20 nm NP	fluorescent dye	27 ± 3	(7.5 ± 0.09) × 10^13^	1.2 × 10^10^
40 nm NP	europium complex	36 ± 1	(6.0 ± 0.01) × 10^12^	2.6 × 10^9^
200 nm NP	fluorescent dye	210 ± 10	(3.9 ± 0.01) × 10^11^	1.0 × 10^7^

^a^Manufacturer's data, ^b^by fluorescence measurement.

For uptake studies, the particles were administered into the isolated intestine, samples from the luminal, vascular and lymphatic compartments were collected over the time course of the experiment and particle concentrations in the different fluids were determined.

**Figure 2:**
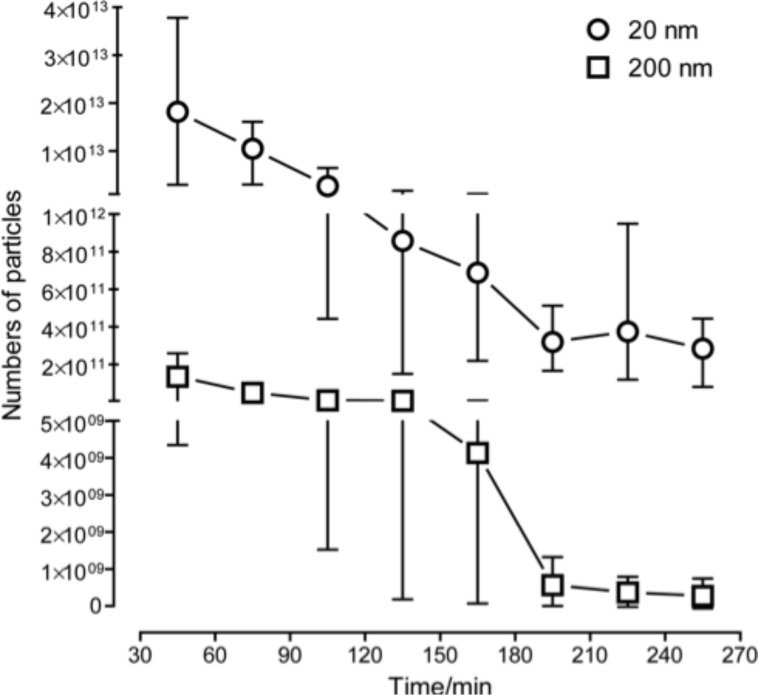
Recovery of nanoparticles in luminal fractions over time. 7.5 × 10^13^ 20 nm NPs or 3.9 × 10^11^ 200 nm NPs were administered at *t* = 30 min. Of all fluids analyzed, only in luminal samples a significant amount of NPs was detected (each *n* = 4, mean with range).

In a first set of experiments 7.5 × 10^13^ 20 nm particles or 3.9 × 10^11^ 200 nm particles were administered into the isolated perfused intestine, and the outflow of the luminal content, the lymph and vascular samples were collected in regular intervals. All samples were analyzed for the presence of NPs by fluorescence measurements. No particle fluorescence could be detected in the vascular and lymphatic samples (data not shown), no matter of which size the particles were. Of all fluids analyzed, only luminal samples contained a significant amount of NPs (Figure 2).

Here, the major amount of NPs was found already in the first fraction, harvested in the first 30 min after application: about 24% (for 20 nm) and 33% (for 200 nm NPs) of the total particle dose administered was detected in these samples. In the following samples the amount of NP decreased constantly. Less than 0.5% of the bolus was found in the final samples (240–270 min). Taking all luminal samples together, only about 40 to 50% of the applied 20 nm and 20 to 70% of the applied 200 nm NPs could be recovered.

**Figure 3:**
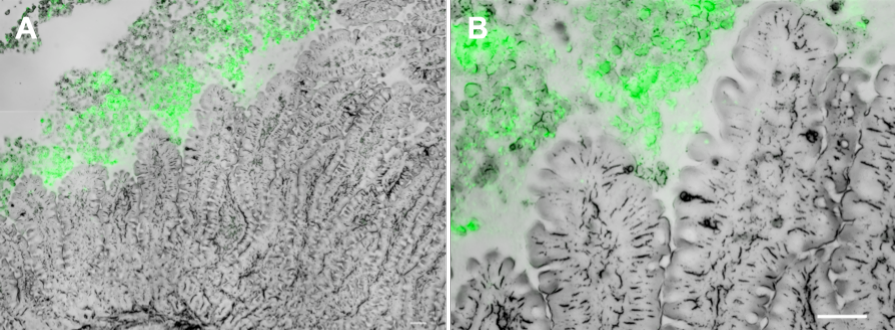
Cryostat sections of gut tissue after application of green fluorescent 20 nm particles. A: overview image, B: excerpt from overview image. Scale bar: 50 µm.

In order to resolve the fate of the missing other half of administered NPs, a histological examination using cryostat sections of the gut (Figure 3) was conducted. It revealed that virtually no particles adhered to the epithelium or resided in the tissue, the bulk of NPs seemed to be trapped in the mucus lining the gut tube.

**Figure 4:**
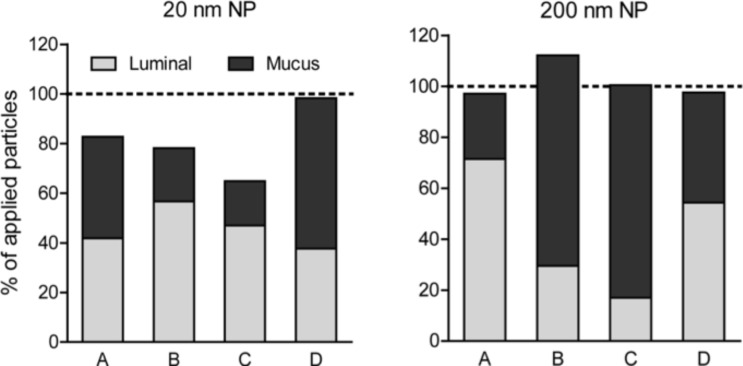
NP recovery from luminal samples and after dissolution of mucus. After collection of luminal samples, the gut mucus was fluidized in situ by a reducing agent, removed from the gut, dissolved, and the particle fluorescence was determined (20 nm and 200 nm NPs, data from four independent experiments (A–D)).

In order to determine the actual amount of mucus-trapped particles, we instilled a reducing agent into the gut lumen after completion of the extracorporeal perfusion experiments. After 20 min incubation time the mucus was sufficiently fluidized to be removed gently from the gut. The mucus was then dissolved completely and the particle fluorescence was determined. With this procedure, almost the entire amount of particles could be recovered: (81 ± 14)% of the 20 nm and (101 ± 8)% of the 200 nm NPs were found in the two fractions, the luminal samples and the dissolved mucus (Figure 4).

**Figure 5:**
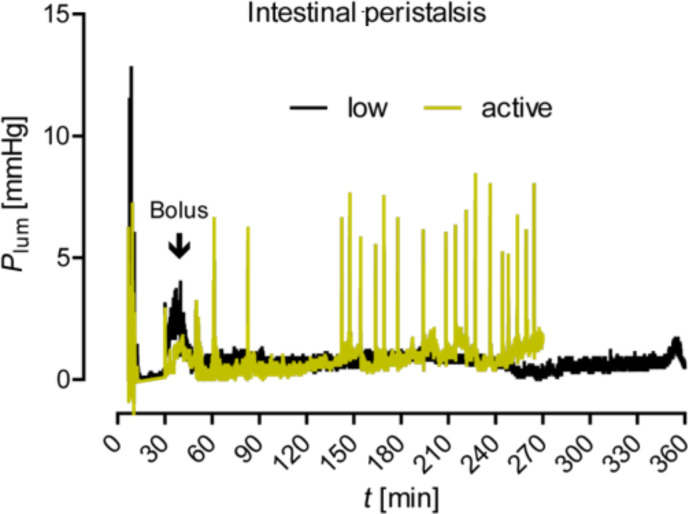
Pressure records of the gut motility with low and active peristalsis.

In this first set of experiments, we had not been able to discover any particles in the lymph and in the vascular system, even though we had applied high amounts of NPs. We therefore decided to modulate our experimental set-up and investigated if an extended epithelial interaction time promotes the uptake of NP. For this purpose we prolonged the duration of the experiment (from 270 to 360 min) and attempted to slow down the intestinal peristalsis by a constant supply of noradrenaline. In these experiments, the mucus was not dissolved by reducing agents, but remained to a large extent in place in the gut tube. As verified by our standard control measurements and histological examinations, the gut viability and tissue integrity remained in the acceptable range under these conditions. The gut motility and intensity of the peristalsis were, as expected, considerably reduced compared to the standard conditions applied before (Figure 5; two short movies, provided in the Supporting Information, illustrate the different peristalsis: M1 high peristalsis, M2 low peristalsis).

**Figure 6:**
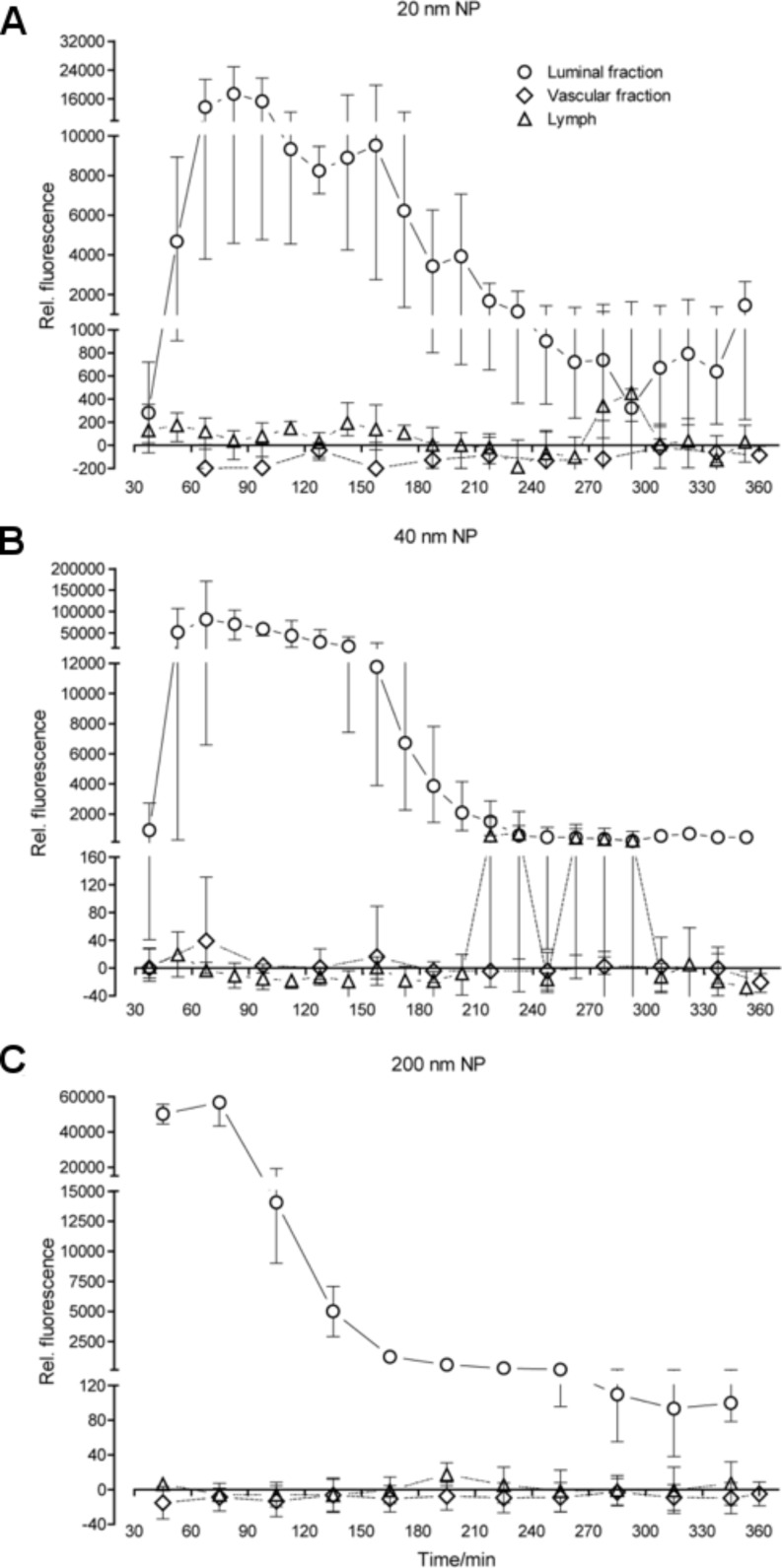
Particle distribution in fluid fractions after extended epithelial interaction time. A: 20 nm NP, B: 40 nm NP, C: 200 nm NP; diamonds: vascular, triangles: lymphatic, circles: luminal samples. The curves represent 2–3 individual experiments (mean with range).

In this “extended contact system”, NPs of 20, 40 or 200 nm diameter again were administered luminally in a bolus (Table 1). Figure 6 shows the particle distribution (A: 20 nm, B: 40 nm, C: 200 nm) in the three fluid fractions, luminal, vascular and lymph.

Even under extended contact conditions, no significant particle fluorescence was detectable in the vascular and lymphatic samples, although some lymphatic samples in one of the three experiments with 40 nm NPs displayed fluorescence signals above background (Figure 6B). As all parameters relating to the gut viability or pressure settings of the luminal and vascular compartment remained in the normal range, no major break-down of the intestinal barrier can be the reason for these signals; at the most, a temporary tiny leakage could be responsible for them. In the luminal fractions, whatever the size of the particles were, the amount of NPs decreased in the course of the experiment, but a baseline level was not reached.

As organic fluorophores can be harmed by cellular products such as reactive oxygen species we had chosen the 40 nm NPs coupled with the rare earth element europium specifically for our experiments so as to be able to determine the presence of particles not only via their fluorescence, but also by measuring the europium content of our samples through an independent procedure. When analyzing our luminal samples by inductively coupled plasma mass spectrometry (ICP-MS), we recovered the europium in concentrations well comparable to the respective fluorescence signals. However, in none of the vascular and lymphatic samples, which we analyzed by ICP-MS, any europium above detection limit was found, not even in the few lymphatic samples where higher fluorescence signals had been measured (Figure 6B).

We then wanted to quantify the particles which still remained in the gut lumen and tissue after completion of the perfusion experiment. Therefore, the gut was sliced in three sections (proximal, medial and distal), the tissue was completely disintegrated, and the europium content was measured by ICP-MS. The highest amount of europium was measured in the distal gut section. Obviously, the bulk of NPs were at first trapped in the mucus and then slowly transported to the end of the gut over the time course of the experiment. However, an overall europium balance with the luminal and tissue samples could not be achieved when using ICP-MS, because the particle amount in some luminal samples was not sufficient to obtain signals above the quantification limit, although in these samples a significant fluorescence signal had been measured. Only (69 ± 14)% (luminal (33 ± 21)%, gut tissue including mucus (36 ± 14)%) of the total particle dose applied could be found when assayed through detection of the europium tracer.

## Discussion

Potential hazards that incorporated NPs may pose to human health are subject of intense debates. A rapidly growing industry hinges on the correct toxicological classification of man-made NPs and the definition of tolerable doses and thresholds for such substances. Crucial for this categorization is the translocation rate with which inhaled or ingested NPs are transported across the mucosal surfaces. Even NPs that are found to be toxic on the cellular and molecular level may not be extremely dangerous if they are intercepted by the body at or before translocation. Yet, with its huge surface area (200–300 m^2^) and the presence of cells that are specialized in particle translocation the human small intestine provides numerous opportunities for NP uptake. On the other hand, features such as digestive enzymes, potentially absorbent constituents of food and flora, mucus and the epithelial glycocalyx as well as the mechanical expulsion by the peristalsis provide protective measures.

In light of the complexity of this setting it is not surprising that a large body of conflicting data exist on the uptake of particles in the small intestine. In rodents, reported in vivo uptake rates range from 2 to 34% of the dose applied for different types of particles, dosages, application protocols and detection regimens [15–17]. Potential reasons for these discrepancies could be, e.g., small injuries during particle instillation, microlesions caused by ingested wooden litter, or differences in the diet and gut flora that influenced NP mobility. In order to minimize such external factors we used the isolated perfused rat intestine where the gut integrity and function are retained but food, intestinal flora and ingested material are washed off before analyzing the interaction of NPs with the small intestine.

A major drawback of this system is the large volume of vascular effluate generated in our setting. Although we changed the protocol from a single-flow to a circulating system, each experiment used about 450 mL of vascular buffer, which is about 30 times the blood volume of our experimental animals (calculated from the body weight and the equation given by [27]). In order to compensate for this dilution factor we decided to use rather high doses of particles (4 × 10^11^ to 7 × 10^13^ per dose), which represents about the 1000-fold amount of NPs that humans ingest per day when normalized on the respective small intestinal surface areas of rat and man [13,28–29]. To locate and quantitate the NPs in tissue and fluid compartments, we chose fluorescent particles, while one type of NPs, the 40 nm europium doped particles, was selected so as to be also measurable, e.g., after acidic tissue disintegration by ICP-MS. This detection method, however, proved to be least sensitive, with a quantification threshold of 2.5 × 10^10^ particles per sample, corresponding to 0.4% of the bolus amount. Due to this rather high detection limit we used the 40 nm europium-doped NPs only in experiments in which the peristalsis was reduced and chances of translocation were considered higher. But even under these conditions it was not possible to detect particles in the lyophilized vascular samples analyzed after completion of the perfusion experiment. With each of these samples comprising about 2–2.5% of the total perfusate volume, the transport of at least 20% of all NPs into the plasma would have been necessary for the europium amount to be quantifiable by ICP-MS. Fluorescence readout was somewhat more sensitive for this particle type, but here also 20% of the bolus would have had to reach the vascular system before NPs could be detected in the samples taken in the time course of the perfusion experiment. Weak fluorescence signals were detected in some lymphatic samples in one challenge experiment with the 40 nm NPs. It is, however, questionable if this represents a true translocation event since neither 20 nm nor 200 nm NPs could be found neither in the vascular nor in the lymphatic compartments although the detection limit of these particles was considerably lower. Translocation of about 1% of the entire 200 nm NPs bolus should have resulted in a measurable fluorescence signal in the vascular samples. In lymph, which amounted to approximately 10–20 mL volume over the course of the experiment, less than 0.1% of the 20 nm- or 200 nm-NPs applied would have been detectable by fluorescence measurements. Yet, as those detection limits were therefore in the range of the reported translocation rates [17] we expected to detect particle translocation under our analytical conditions. The fact that we did not observe such events, however, does not allow for the claim that NP translocation did not happen at all. In light of the somewhat inconsistent particle balances in the luminal outflow samples, NP uptake may well have occurred, even if we could not demonstrate the presence of particles in either circulation, lymph, nor tissue. Thus, we could not resolve the controversy about the different particle translocation rates in the gut with our model system. We could, however, shed light on another important player that appears to dramatically interact with the NPs tested, the mucus.

In previous studies rats were often challenged over a certain time interval with several particle doses before sacrifice. Particles taken-up were then determined after dissecting and thoroughly washing the intestine [15–17]. Consequently, all processes occurring between ingestion and tissue deposition could not be monitored and hence were ignored, including any interactions between NPs and the mucus. With our histological analysis performed on intestinal tissue after the ex vivo NP challenge experiments we could show that the bulk of the particles was trapped in the mucus layer and only few particles adhered or were taken up. This led us to speculate that the mucus acts as a major particle trap. This hypothesis is supported by our finding that, based on the fluorescence measurements, about 50% of the NPs were "lost" in the explanted rat intestine when the mucus layer was not removed, whereas fluidization of the mucus layer by a reducing agent allowed recovery of about 100% of the 200 nm NPs and an average of 80% of the 20 nm NPs. Whether the missing 20% of the latter were actually taken up by the gut tissue and disseminated into the lymph and into the artificial blood remained unclear. Also, the various fluidic components of our system may have exerted a certain influence on the fluorescence of the particles. We therefore checked for this effect but found no differences between particles suspended in either fresh luminal buffer, luminal outflow, vascular buffer or lysis buffer. Merely the presence of high concentrations of the reducing agent TCEP reduced the fluorescence of 200 nm particles between 10 and 30% (depending on particle concentration), albeit no reduction was found with the 20 nm particles. Since our fluorescence measurements had indicated a virtually complete recovery for the 200 nm particles from combined luminal and dissolved mucus samples, we deemed the potential fluorescence-quenching effect of the reducing agent not to be substantial in our setting.

In general, histological examination showed only a very small fraction of the NPs to be attached to the epithelium after removal of the mucus. Our data therefore lead us to the conclusion that the mucus is a major if not the most important player protecting the epithelium against particle uptake, and if it cannot completely abolish NP translocation it will at least greatly reduce it. This fits well with the role of mucus as an essential component of the innate immune system [30–31]. Yet, its protective functions must not be seen as a static condition, but alterations in mucus production and composition have been shown to occur in response to microbial challenges, variations in dietary constituents and in inflammatory intestinal disorders [30,32–34]. In light of this, it should be interesting to learn how the intestinal mucosa deals with NPs in times of disturbed mucus production. Combining all information presently available, we must assume that especially inflammatory conditions at the intestinal mucosa carry an increased risk of NP uptake.

## Conclusion

In conclusion, after administration of a high dose of NPs into a rat perfused intestine the vast majority of NPs did not come into contact with the epithelium but was either directly discarded from the gut or trapped in mucus. On the basis of our findings we suppose that a healthy small intestinal tract provides an effective barrier against NP uptake whereby the mucus film seems to be a central protagonist.

## Experimental

### Animals, dissection and perfusion technique

All animal experiments were approved by the local authorities (Ministry of Agriculture, Environment and Rural Areas of the State of Schleswig-Holstein, Kiel, Germany). Non-fasted female Wistar rats (Charles River Laboratories, Sulzfeld, Germany) with a mean weight of 249 ± 19 g were used as donors.

The dissection and cannulation of the small intestine, the layout of the perfusion chamber (Hugo Sachs Elektronik-Harvard Apparatus, March-Hugstetten, Germany; see Figure 1) and the perfusion of the intestinal explant were performed as described by Lautenschläger et al. [26] with the following modifications: The vascular perfusate was recirculated under permanent supplementation of oxygen at the arterial inlet, with a total volume of artificial blood plasma not exceeding 450 mL in one perfusion experiment. Because of this switch from single-path perfusion to recirculation, the concentration of noradrenaline (norepinephrine hydrochloride, Sanofi-Aventis, Frankfurt, Germany) in the artificial blood plasma was increased to 0.122 mg/L (i.e., 54.9 µg/450 mL). For experiments with reduced gut motility, a constant noradrenaline supply of 54.9 µg per hour was given additionally from minute 40 until the end of the experiment. The luminal and vascular flow rates, explant weight, as well as arterial, venous, and luminal pressures were continuously recorded. The vascular perfusate was analyzed at regular intervals for O_2_ and CO_2_ partial pressures, pH, electrolytes, glucose, and lactate [26].

### Nanoparticle challenge experiments

A continuous luminal perfusion of the explant with a flow rate of 3 mL/h for 30 min was used to equilibrate the whole system. After equilibration, the NPs were administered in a total volume of 1 mL, and for 10 min the luminal flow rate was increased to 6 mL/h in order to forward the NPs fast into the explant. After this time, the flow rate was again adjusted to 3 mL/h. The outflow of the luminal content and the lymph were collected over periods of 15 and 30 min each for a total time of 270–360 min. Vascular samples were taken every 30 min from the recirculating perfusate.

To collect particles still entrapped in the gut after termination of the perfusion, the intestinal mucus was removed and dissolved. To do so, the luminal outflow side of the intestine was reversibly closed with a ligature, and 1.5 mL 50 mM TCEP (tris-(2-carboxyethyl)phosphine hydrochloride, C. Roth, Karlsruhe, Germany) in water were instilled in the inflow opening. After 20 min incubation the luminal outflow side was reopened, and the luminal contents were harvested by first flushing the gut with air and then gently squeezing the tissue with moist cotton pads. Mucus was dissolved in a lysis buffer (100 mM Tris-Cl, pH 8.0–8.5, 200 mM NaCl, 0.2% SDS, 5 mM EDTA, 100 µg/mL proteinase K) for 15 to 30 min, and the fluorescence of the solubilized effluate was determined.

### Nanoparticle quantitation

Fluorescent, carboxylate-modified polystyrene NPs (FluoSpheres^®^, Table 1) were purchased from Invitrogen (via Life Technologies; Darmstadt, Germany). The fluorescence of the 20 nm and 200 nm NPs was directly measured in suspensions (standards and samples) by using a multi-mode microplate reader (SpectraMax M5; Molecular Devices, Biberach an der Riss, Germany) at excitation/emission wavelengths of 480/515 nm. The luminescence of the europium-containing 40 nm NPs was analyzed time resolved with a delay of 200 µs and an integration time of 1000 µs with excitation/emission wavelengths of 360/610 nm. The measured fluorescence/luminescence of every sample was transformed to particle numbers by comparison with a standard curve. Control experiments showed now alteration in the measured fluorescence when particles were suspended in any of the different solutes analyzed. Only addition of 50 mM TCEP to the 200 nm particles resulted in a decrease in fluorescence of 10 to 30%, depending on the particle concentration.

### ICP-MS

Tissue samples for ICP-MS analysis were lyophilized (150–370 mg dry weight), lymph and luminal outflow samples (130–1000 µL) were used as collected. Portions of vascular buffer were freeze-dried after completion of perfusion experiments, and aliquots corresponding to 2–2.5% of the total perfusate were analyzed. The weighed samples were mixed with 2 mL ultrapure water and 1 mL concentrated nitric acid, dissolved by microwave treatment and adjusted to a volume of 5 mL. An internal standard (rhodium, Rh) was added to every sample. The mass of europium ^153^Eu and internal standard ^103^Rh was measured with a PE-Elan DRC II ICP-MS, the amount of europium in the samples was derived from a standard curve, established with Eu standard solution (Bernd Kraft GmbH, Duisburg, FRG) over a measurement range of 0.1 to 10 µg/L (limit of quantification 250 ng/sample). A stock solution of 40 nm NPs of known concentration was analyzed accordingly to correlate the particle numbers with the Eu mass.

### Histological examinations

For post-perfusion analysis of the gut integrity, paraffin embedded tissue sections (5 µm) were stained with hematoxylin and eosin. The histological stability score was determined by comparing the number of intact villi to the total number of villi in randomly selected slices [26]. After perfusion additional small tissue sections from the explant were embedded in tissue freezing medium (Tissue-Tek^®^ O.C.T.™, Sakura, Staufen, Germany) and frozen in liquid nitrogen. Cryostat sections with a thickness of 5 µm were analyzed microscopically for the presence and distribution of particle fluorescence.

## Supporting Information

Supporting Information features video recordings of the gut mobility. For the offline video analysis of the intestinal peristalsis, a standard digital miniature camera mounted on the lid of the explant chamber continuously filmed the gut. Overall motility was monitored in all sections of the isolated organ. The luminal pressure fluctuations correlated well with the visual observation of peristalsis.

File 1 Video recording of high intestinal peristalsis after 120 min perfusion and luminal administration of 200 nm NPs.

File 2 Video recording of low intestinal peristalsis, due to constant supply of noradrenaline, after 270 min perfusion and luminal administration of 200 nm NPs.
